# Acupuncture May Decrease the Incidence of Post-stroke Dementia: A Taiwan Nationwide Retrospective Cohort Study

**DOI:** 10.3389/fneur.2021.657048

**Published:** 2021-05-21

**Authors:** Shao-Ang Chu, Te-Yuan Chen, Po-Yuan Chen, Wei-Jie Tzeng, Cheng-Loong Liang, Kang Lu, Han-Jung Chen, Cheng-Chun Wu, Jian-Han Chen, Chin-Chuan Tsai, Hao-Kuang Wang

**Affiliations:** ^1^Department of Neurosurgery, E-Da Hospital, Kaohsiung, Taiwan; ^2^School of Medicine, College of Medicine, I-Shou University, Kaohsiung, Taiwan; ^3^Department of General Surgery, E-Da Hospital, Kaohsiung, Taiwan; ^4^Chinese Medicine Department, E-Da Hospital, Kaohsiung, Taiwan; ^5^School of Chinese Medicine for Post-Baccalaureates, I-Shou University, Kaohsiung, Taiwan

**Keywords:** acupuncture, dementia, stroke, incidence, outcome

## Abstract

**Background:** Post-stroke dementia may affect up to one-third of stroke survivors. Acupuncture as a complementary treatment for stroke has been shown to be beneficial for subsequent post-stroke rehabilitation. The purpose of this retrospective cohort study was to investigate the potential effect of acupuncture to protect stroke patients from dementia.

**Methods:** We included 9,547 patients receiving ambulatory or hospital care for stroke and 9.547 non-stroke patients; patients were matched for sex, age, and Charlson Comorbidity Index. Each individual was traced for the subsequent development of dementia. Two thousand four hundred and forty-nine stroke patients received acupuncture treatment and 7,098 residue stroke patients without acupuncture treatment served as control groups. This is a 3-year follow-up cohorts study: the incidence and adjusted hazard ratios (HRs) with 95% confidence intervals (CIs) of post- stroke dementia in the Cox proportional hazard regression.

**Results:** During the 3-year follow-up, 1,403 patients with stroke (14.70%) and 427 patients without stroke (4.47%) developed dementia. The adjusted HRs of development of dementia among stroke patients were 3.64-times (range, 3.27–4.06), and the incidence of dementia was higher in male. Stroke patients receiving acupuncture treatment had a lower probability of dementia than those without acupuncture during the follow-up period, the adjusted HRs was 0.49 (95% CI, 0.42–0.58; *p* < 0.001).

**Conclusions:** The association between stroke and dementia existed in both sexes, more prominent in male. Patients with stroke receiving acupuncture treatments showed decreased risk of dementia. Care must be taken evaluating these results because this study was limited to lack of information regarding lifestyles, stroke severities, and acupuncture methods that were used in treatments.

## Background

Stroke has been recognized as one of the major causes of adult disability worldwide. Survivors of stroke are at increased risk of developing cognitive impairment due to acute tissue damage. Assuredly, one-third of these survivors are found to have a significant degree of cognitive impairment within the first month after the event (1–3). The lifetime risk of developing either stroke or dementia by age of 65 is one in three in men and one in two in women (4). Post-stroke condition constitutes a well-defined reason of dementia first recognized by Sir Thomas Willis in 1672 under the name post-apoplectic dementia (2). This is an etiological classification of dementia characterized by cognitive impairment as a result of ischemic or haemorrhagic stroke. Hypoperfusion and thromboembolism events lead to decreased cerebral blood flow, hypoxia, and oxidative stress and trigger inflammatory responses, subsequently damaging the area of brain important for memory, cognition, and behavior (5). Although functional recovery develops over the course of 26 weeks after a stroke, and physical impairments tend to improve to a greater or lesser degree, the survivors are often left with disabilities and worsening cognitive impairments (6, 7).

Currently there is no definitive and effective treatment for post-stroke dementia. Treatments with antioxidants, anti-inflammatory agents or agents increasing cerebral perfusion have not provided satisfactory results (8). Numerous clinical studies and systemic reviews support the therapeutic effects of acupuncture in vascular dementia (VD) and post-stroke rehabilitation (9–11). However, these therapeutic effects remain unclear because of poor methodology and small sample sizes (12, 13). Acupuncture, with many categories such as manual acupuncture, electroacupuncture, laser acupuncture, and acupoint injection, serves as a form of alternative treatment and an essential component of traditional Chinese medicine which can be traced back 3,000 years (9). It is a relatively safe procedure with few adverse effects. The mechanisms of acupuncture on VD are still uncertain; however, animal-based studies demonstrate the possibility of reducing oxidative stress, attenuating neuronal apoptosis, relieving neuroinflammation, regulating glucose metabolism, modulating neurotransmitters, and improving synaptic plasticity and blood vessel function (14). Acupuncture has become universally accepted. The 2007 US National Health Interview Survey found 3.1 million adults and 150,000 children had received acupuncture in the previous year, and acupuncture use increased overall between 2002–2007 (15, 16).

It is unknown if acupuncture is an adequate therapeutic method to reduce post-stroke dementia. Using the Taiwan National Health Insurance Research Database(NHIRD), we conducted a retrospective, nationwide population-based cohort study to investigate the possibility of acupuncture use in reducing incidence of post-stroke dementia.

## Methods

### Database

The study was approved by the E-Da hospital Ethics Review Committee, Taiwan (EDA-JIRB-2014012). We conducted a retrospective, population-based cohort study of patients who are registered in the Longitudinal Health Insurance Database (LHID2000). 98.4% of the population in Taiwan (~22.96 million) has been enrolled in Taiwan's NHIRD, which comprises healthcare data from the medical records of all beneficiaries (18, 19). The LHID2000 is composed of 1 million insured subjects randomly selected from the NHIRD without bias to age and gender. The International Classification of Diseases, Ninth Revision, Clinical Modification (ICD-9-CM) diagnostic and procedure codes (established according to the World Health Organization criteria) are included in the diagnostic data (17). Details of the generation, monitoring, and maintenance related to the database are published online by the Taiwan's National Health Research Institutes (http://nhird.nhri.org.tw/) (18, 19).

### Study Sample

**Figure 1:**
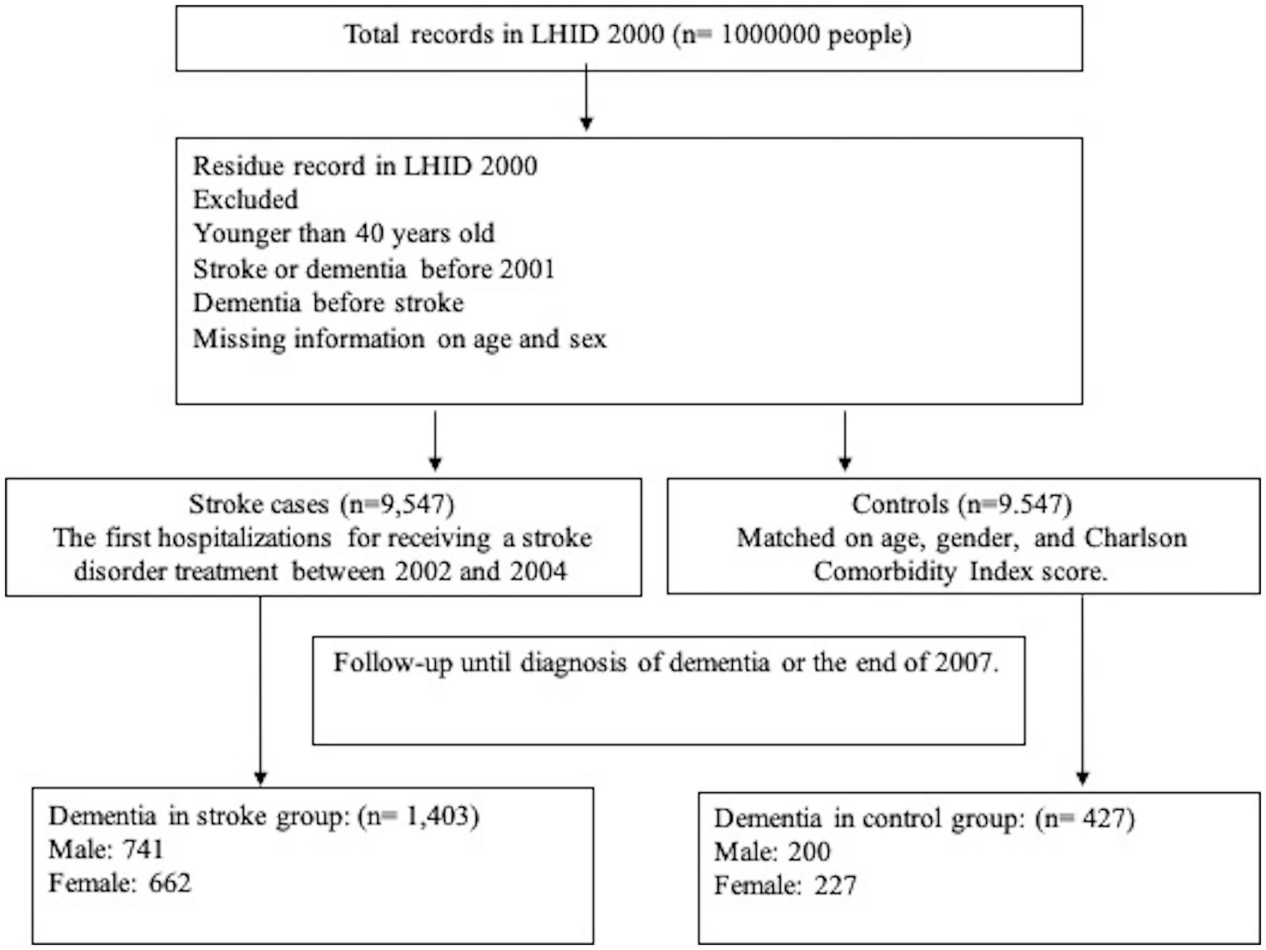
Flow chart of the study population.

Patients with a diagnosis of stroke (ICD-9-CM code 430-437), receiving treatment in the hospital between January 1st, 2002 and December 31st, 2004 were enrolled in this study (18). A flow chart of this study is shown in Figure 1. We excluded patients aged younger than 40 to restrict the study to the assessment of the most common etiology of stroke. In order to confirm that all patients with stroke enrolled in this study were incident cases, only new-onset stroke patients were included. Patients with the diagnosis of stroke or dementia before 2001 were excluded. Patients with stroke without acupuncture treatment were selected by a matched pair procedure with propensity score (exposure vs. non-exposure ratio = 1:1) by age, gender, and Charlson Comorbidity Index. Furthermore, patients who had the following conditions prior to the initial use of health care facilities were identified and excluded from this study: ICD-9-CM code 290.0 (senile dementia, uncomplicated); 290.1 (presenile dementia); 290.2 (senile dementia with delusional or depressive features); 290.3 (senile dementia with delirium); 290.4 (arteriosclerotic dementia); 294.1 (dementia in conditions classified elsewhere); 331.0 (Alzheimer's disease, AD); 331.1 (Pick disease); and 331.2 (senile degeneration of the brain). A total of 9,547 subjects who had strokes from 2002 to 2004 were matriculated in this study.

A comparison group was randomly extracted from the records of the remaining patients in the database with identical exclusion criteria. Then the controls were matched 1: 1 for sex, age, and Charlson Comorbidity Index score.

### Main Outcome Measures

Each patient was individually tracked for 3 years from their index use of the healthcare facilities to identify those who subsequently suffered incident dementia [International Classification of Diseases, Ninth Revision, Clinical Modification (ICD-9-CM) codes: 290.0, 290.1, 290.2, 290.3, 290.4, 294.1, 331.0, 331.1, and 331.2] We included only cases that consisted of at least 3 NHI ambulatory-claim records, or 1 inpatient record, in order to increase the validity of dementia diagnosis. Then, acupuncture (procedure code: B41, B42, B45, B46, P27041, P33031) data at least 3 NHI ambulatory-claim records was collected to determine whether it is an effective treatment in reducing the incidence of post-stroke dementia. Some patients receive combination therapy with acupuncture and Chinese herbs were enrolled in our study. We also categorized the data for these dementia subtypes; AD (ICD-9-CM code 331.0), VD (ICD-9-CM code 290.4), and unspecified dementia (ICD-9-CM codes 290.0–290.4, 294.1, and 331.1–331.2).

### Statistical Analysis

Cases and controls were followed from the initial hospitalization date until December 31st in 2007. Pearson's chi-square-test was administered to compare the baseline characteristics of the stroke cases receiving acupuncture and non-stroke controls from the NHIRD. Selected comorbidities were included if they occurred in either an inpatient setting or 3 or more ambulatory care claims that had been recorded 1 year before the initial ambulatory care visit. Multivariate Cox proportional hazard regression model was conducted to evaluate the hazard ratios (HRs) with 95% confidence intervals (CIs) for risk of dementia in patients receiving acupuncture after occurrence of stroke. Age, gender, and selected comorbidities were adjusted. Apart from the abovementioned comorbidities, we also adjusted for acupuncture in the regression modeling. A *p*-value < 0.05 was defined as statistical significance in this study. The 5-years disease-free survival rate was evaluated with the Kaplan-Meier log rank test to examine differences between cohorts. A statistical analysis software system (SAS, System for Windows, version 9.2, SAS Institute, Inc., Cary, NC, USA) was used to perform the statistical analyses.

## Results

Overall, 19,094 patients were included in the stroke and non-stroke cohorts, respectively. There were no significant differences in age or gender between these groups. Geographic characteristics, comorbidities and medication usage of the patients with stroke and comparison patients are shown in Table 1. The proportion of men was higher than that of women in both cohorts (53.48 vs. 46.52%). The mean age of the patients was 65.90 ± 11.66 years. After matching for age, gender, and Charlson Comorbidity Index score. the comorbidities of diabetes, hypertension, coronary heart disease, heart failure, atrial fibrillation, peripheral vascular disease, respiratory system, peptic ulcer disease, chronic liver disease, chronic kidney disease, rheumatologic disease, and cancer were more prevalent in the stroke cohort, compared with the non-stroke cohort. Use of medications in this study, such as antihypertensive agents, and lipid-lowering agents revealed no statistically significant between two cohorts.

**Table 1:** Geographic characteristics and comorbidities of the patients with stroke and comparison patients.

	**Patients with stroke**		**Comparison patients**		
	**(*****N*** **=** **9,547)**		**(*****N*** **=** **9,547)**		
**Characteristic**	***N***	**%**	***N***	**%**	***P***
Gender					0.8618
Male	5,106	53.48	5,118	53.61	
Female	4,441	46.52	4,429	46.39	
Age, mean ± SD	65.90 ± 11.66		66.02 ± 11.72		0.4808
Age group					0.9658
40–49	1,017	10.65	1,029	10.78	
50–59	1,781	18.66	1,786	18.71	
60–69	2,626	27.51	2,640	27.65	
70–79	2,995	31.37	2,996	31.38	
≥80	1,128	11.82	1,096	11.48	
CCI, mean ± SD	3.13 ± 2.01		3.11 ± 1.97		0.5978
Comorbidities					
Diabetes	3,157	33.07	3,762	39.41	<0.0001
Dyslipidemia	16	0.17	9	0.09	0.1612
Hypertension	7,189	75.3	4,970	52.06	<0.0001
Coronary heart disease	3,317	34.74	2,664	27.9	<0.0001
Heart failure	879	9.21	1,111	11.64	<0.0001
Atrial fibrillation	399	4.18	244	2.56	<0.0001
Peripheral vascular disease	874	9.15	701	7.34	<0.0001
Respiratory system	2,308	24.18	3,286	34.42	<0.0001
Peptic ulcer disease	3,069	32.15	4,619	48.38	<0.0001
Chronic liver disease	2,004	20.99	3,552	37.21	<0.0001
Chronic kidney disease	1,160	12.15	1,946	20.38	<0.0001
Rheumatologic disease	449	4.7	651	6.82	<0.0001
Cancer	638	6.68	1,856	19.44	<0.0001
**Medication usage**					
Antihypertensives	4	0.04	2	0.02	0.4141
Lipid-lowering agents	8	0.08	7	0.07	0.7962

*Chi-square-test and t-test*.

**Figure 2:**
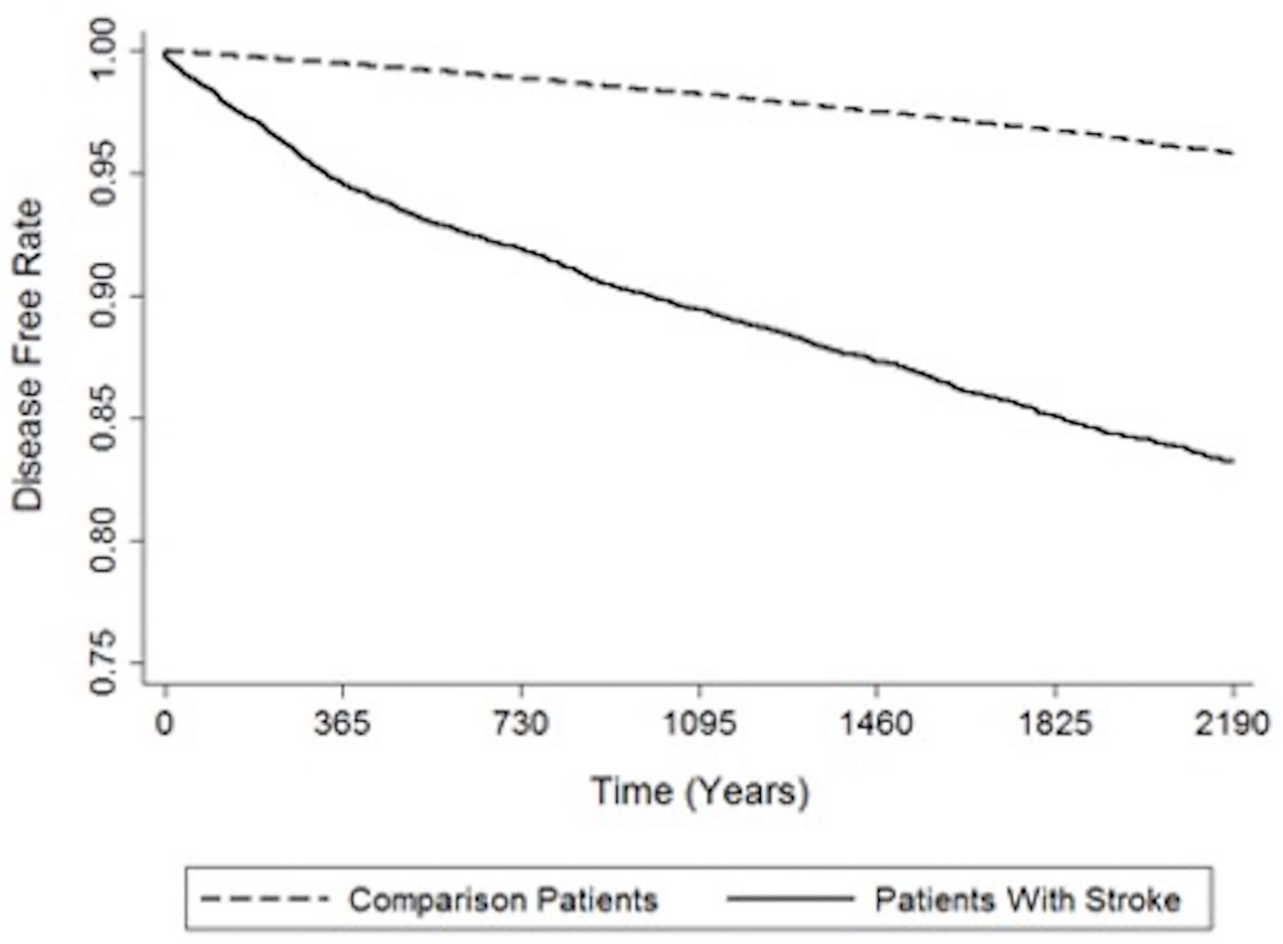
Indicates the disease-free survival curves determined using the Kaplan–Meier log rank analysis.

The crude and adjusted HRs of dementia among patients and controls in each gender group during a 3-year follow-up period from initial treatment are shown in Table 2. Compared to the patients in the control cohort, patients with stroke had significantly higher incidence of dementia (14.70 vs. 4.47%). The values of stroke patients were higher than corresponding values in the non-stroke cohort; 3.64 (95% CI, 3.27–4.06; *p* < 0.001) experienced dementia within the follow-up period according to Cox proportional hazard regressions (stratified by gender, age group, and the year of initial health care). We further analyzed the HRs according to gender after adjusting the data for the presence of comorbidities. The HRs acquired within the male group and female group were 4.12 (95% CI 3.51–4.80, *p* < 0.001) and 3.24 (95% CI 2.779–3.77, *p* < 0.001), respectively. Figure 2 indicates the disease-free survival curves determined using the Kaplan–Meier log rank analysis.

**Table 2:** Crude and adjusted HRs of dementia among patients and controls in each gender group during a 3-year follow-up period from initial treatment.

	**Patients with stroke**		**Comparison patients**	
	***N*** **=** **9,547**		***N*** **=** **9,547**	
**Dementia occurrence**	***N***	**%**	***N***	**%**
**Total**	1,403	14.70	427	4.47
Crude HR (95% CI)	3.565 (3.199–3.973)		<0.0001	
Adjusted HR (95% CI)	3.640 (3.266–4.057)		<0.0001	
**Male**	741	14.51	200	3.91
Crude HR (95% CI)	4.046 (3.461–4.731)		<0.0001	
Adjusted HR (95% CI)	4.109 (3.514–4.804)		<0.0001	
**Female**	662	14.91	227	5.13
Crude HR (95% CI)	3.141 (2.701–3.652)		<0.0001	
Adjusted HR (95% CI)	3.239 (2.785–3.766)		<0.0001	

*Adjusted for age, gender, geographic region, medication usage, and Charlson Comorbidity Index*.

**Figure 3:**
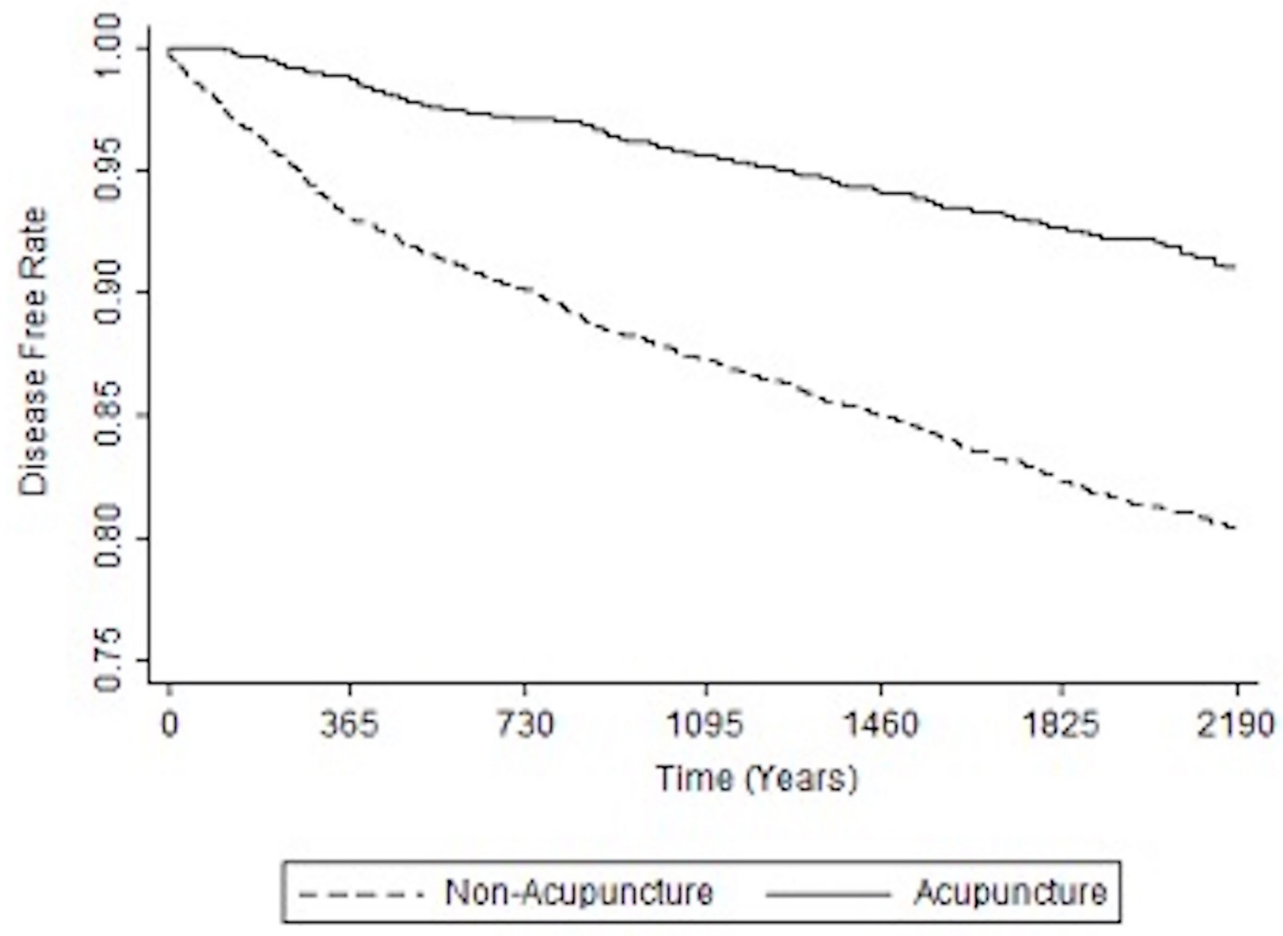
Kaplan-Meier curve of the estimated dementia-free proportions of patients with stroke who received and did not receive acupuncture treatment.

**Figure 4:**
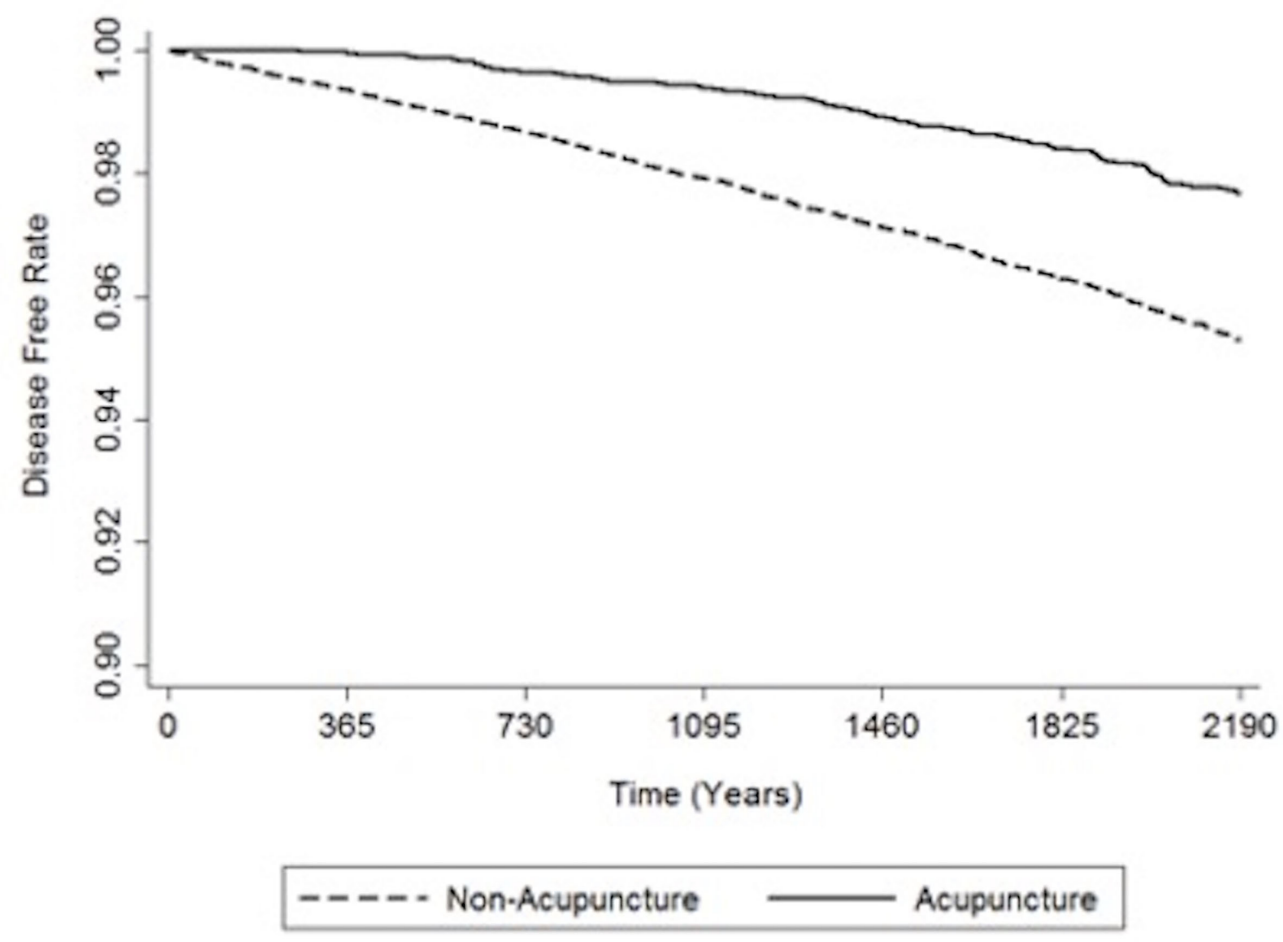
Kaplan-Meier curve of the estimated dementia-free proportions of non-stroke patients who received and did not receive acupuncture treatment.

Table 3 shows the relative risk of developing distinct types of dementia after acupuncture treatment in the stroke patient group. The analysis consistently demonstrates that, compared to stroke patients without acupuncture, the study patients were less likely to sustain several subtypes of dementia during the 3-year follow-up period after their initial assessment. Notably, the adjusted HRs for other dementia in patients with acupuncture was 0.48 (95% CI, 0.40–0.58; *p* < 0.001). Figures 3, 4 disclose the disease-free survival curves of the patients with stroke and comparison cohorts who did and did not undergo acupuncture using the Kaplan–Meier method.

**Table 3:** Relative risk of developing distinct types of dementia after acupuncture treatment in the stroke patient group.

	**Patients with acupuncture**		**Non-acupuncture**	
	***N*** **=** **2,449**		***N*** **=** **7,098**	
	***N***	**%**	***N***	**%**
**Total**	183	7.47	1220	17.19
Crude HR (95% CI)	0.399 (0.341–0.466)		<0.0001	
Adjusted HR (95% CI)	0.491 (0.420–0.575)		<0.0001	
**Alzheimer disease**	7	0.29	61	0.86
Crude HR (95% CI)	0.304 (0.139–0.665)		0.0028	
Adjusted HR (95% CI)	0.395 (0.180–0.871)		0.0213	
**Vascular dementia**	41	1.67	216	3.04
Crude HR (95% CI)	0.503 (0.360–0.702)		<0.0001	
Adjusted HR (95% CI)	0.574 (0.409–0.804)		0.0012	
**Other dementia**	138	5.63	956	13.47
Crude HR (95% CI)	0.384 (0.321–0.459)		<0.0001	
Adjusted HR (95% CI)	0.482 (0.402–0.577)		<0.0001	

*Adjusted for age, gender, geographic region, medication usage, and Charlson Comorbidity Index*.

The crude and adjusted HRs of dementia among patients with stroke and those who have received acupuncture in this study are shown in Table 4. Adjusted HR of dementia for patients with stroke was 4.73 (95% CI 3.34–4.15, *p* < 0.001), and surprisingly, the result reveals that patients who received acupuncture, whether they have a history of stroke or not, are at lower risk of developing dementia during the 3-year follow-up.

**Table 4:** Crude and adjusted HRs of dementia among patients with stroke and those who have received acupuncture.

	**Crude (95% CI)**	**Adjusted HR (95%CI)**
**Total**		
Stroke	3.670 (3.293–4.090)^*^	3.723 (3.340–4.150)^*^
Acupuncture	0.421 (0.368–0.482)^*^	0.539 (0.470–0.619)^*^

*Regression Crude: independent var-Stroke and Acupuncture*.

*Regression Adjust: independent var- Stroke, Acupuncture, Age, Gender, Geographic region, medication usage, and Charlson Comorbidity Index*.

* *p < 0.0001*.

## Discussion

The consequence of this retrospective, nationwide, population-based cohort study illustrates significant associations between post-stroke dementia and application of acupuncture. The best methods to prevent post-stroke dementia are to impede recurrence of stroke and reduce the severity of stroke-related impairment through the most favorable acute treatment and intensive secondary prevention (20–22). Secondary prevention consists of medical interventions and lifestyle modification. To date, advanced treatment for vascular risk factors and vascular disease may prevent post-stroke dementia. An observational study unveiled reduced cognitive impairment in patients with appropriate post-stroke vascular risk management, including antiplatelet therapy, antihypertensive drugs and statins, or anticoagulants, as indicated (21). Furthermore, cognitive function can benefit from therapy for neuropsychiatric symptoms like depression, apathy, and anxiety, as well as cognitive training/stimulation (22). According to our results, we suggest that early intervention with acupuncture could also decrease the risk of post-stroke dementia.

Acupuncture as a traditional Chinese healing technique treats disorders by inserting needles into specific acupoints, and it is generally considered safe when performed correctly. The use of acupuncture treatment for 43 diseases has been recommended by the World Health Organization (23), and as a non-pharmacological intervention, it is considered to be an alternative treatment for dementia (10–14). In a recent animal study, improvement in cognition and hippocampal synaptic plasticity induced by acupuncture was achieved *via* activation of dopamine D1/D5 receptors (24).

After adjusting for geographic characteristics, region of residence, and selected comorbidities, medication usage, the proportion of men with diagnosis of stroke was a bit higher than that of women in both cohorts (53.48 vs. 46.52%) during this 3-year follow-up. The selected comorbidities were more prevalent in the stroke cohorts compared with the non-stroke cohorts. Use of medications revealed no statistically significant between two cohorts. The mean age of the patients in the cohorts was 65.90 ± 11.66 years. In another study, older age, residents in southern and eastern regions, patients with low insurance range, and antiplatelet use were prominent risk factors of post-stroke dementia in Taiwan (25).

We demonstrated that patients with stroke had significantly higher incidence of dementia rates within a 3-year follow-up, and the incidence of dementia in male patients with stroke was higher than that of female patients with stroke. A pooled analysis of international data classified important contributors and risk factors to post-stroke dementia, and age over 65 and female gender are the mentioned risk factors (26, 27). The mean age in our study and the result that stroke patients had higher incidence of dementia match with this analysis, however the effect of gender did not. Although there was no statistical significance, the proportion of male patients with diagnosis of stroke in our study was higher. Li et al. claimed that patients with stroke were at significantly more risk of dementia during the 5- and 10-year follow-up periods in Taiwan (25). Post-stroke dementia is often recognized in the first weeks to months post-ictus, and thereafter, prevalence of post-stroke dementia increases with time (28). Ethnic differences and a longer follow-up period should be considered.

The relationship between stroke and neurodegenerative diseases, such as AD and VD, has been established (1–5). VD is the second most common cause of cognitive decline, with only AD been more prevalent (29). Stroke patients with prior atherosclerotic cardiovascular disease are more likely to suffer from subsequent dementia (30). Risk of dementia is probably related to individual clinical characteristics, it could also depend on the acute lesion (e.g., severity, location, sub-type), pre-existing burden of lesion, vascular risk factors, and other determinants of susceptibility, such as different level of education, white matter disease, and previous cognitive function. Variation in dementia risk was substantial, the 5-year cumulative incidence ranged from 0% after transient ischemic attack in patients aged younger than 65 years to more than 80% in those aged 75 years or older with the diagnosis of major recurrent stroke. This highlighted the essential of individualized prognostication (31). Multicellular interactions within the neurovascular unit, including damage to the blood-brain barrier, neuronal cell death or degeneration, glial reaction, and immune cell infiltration contribute to the etiology (32). Our results indicated stroke patients with subsequent acupuncture treatment were less likely to develop several subtypes of dementia during the 3-year follow-up. Notably, the adjusted HR for unspecified dementia in patients with acupuncture was 0.48 (95% CI, 0.40–0.58; *p* < 0.001). Pathological mechanisms of AD are poorly understood, and ~70% of the risk is believed to be inherited, with many genes being involved (33). VD can also result from other conditions besides stroke that damage blood vessels and reduce circulation. This may explain why these most common etiologies of dementia did not show statistical significance in our study, although there was a trend toward affirmative efficacy.

There is no obvious evidence to support any specific mechanism that contributes to the effect of acupuncture in decreasing the incidence of post-stroke dementia. Researchers have discovered that this therapy may reduce oxidative stress, attenuate neuronal apoptosis, relieve neuroinflammation, regulate glucose metabolism, modulate neurotransmitters, and improve synaptic plasticity and blood vessel function (14). Interestingly, we discovered evidence that revealed that those patients who received acupuncture were at a lower risk of developing dementia regardless of whether they had the diagnosis of stroke or not. Patients who choose acupuncture as a complementary treatment may place more emphasis on personal health maintenance with regard to better knowledge and attitudes for disease prevention. These factors could also contribute to reduce the incidence of dementia.

A retrospective, nationwide cohort was employed in our study, which provided a large sample size and more evidence compared to case-control or cross-sectional study designs. Problems like insufficient power and the effect of selection biases were minimized. However, our study had several limitations. First, the insurance claims data we collected lacked information regarding clinical risk scores (such as the National Institutes of Health Stroke Scale or the Barthel Index), characteristics of the lesion, its location, size, laboratory examination results, and factors of patients' lifestyle such as cigarette smoking, alcohol consumption, and physical activity. Second, this study could not validate the actual acupoints used in the treatment of the stroke patients, as limited information is provided by the National Health Insurance Research Database. The acupoints chosen by traditional Chinese medicine physicians for stroke patients vary, and the beneficial effects are somewhat different (34). Further studies need to be carried out to identify the protective effects against dementia from individual acupoints. Third, it is well-known that smoking, and body mass index are very important risk factors for dementia. This study lacks information regarding the early recognition those risk factors (35, 36). Finally, residual confounding data is always present, even though we had utilized multivariate regression adjustments for several potential confounding factors in our analysis.

## Conclusions

From the results of this nationwide retrospective cohort study, stroke patients with subsequent acupuncture treatment have a significantly lower risk of post-stroke dementia after adjustments for selective confounding factors. The beneficial effect of acupuncture was investigated in our study, yet still very little is known regarding the mechanisms of acupuncture. Care must be taken in extrapolating from our results, and further investigations that overcome the limitations of this study are essential to ascertain the mechanisms for such effects and provide more evidence of the association.

## Data Availability Statement

Publicly available datasets were analyzed in this study. This data can be found here: Taiwan's National Health Research Institutes (http://nhird.nhri.org.tw/ accession number: EMRP-108-061).

## Ethics Statement

The studies involving human participants were reviewed and approved by E-Da hospital, EMRP-108-061. Written informed consent for participation was not required for this study in accordance with the national legislation and the institutional requirements.

## Author Contributions

S-AC, C-CT, C-CW, and H-KW designed research. S-AC, T-YC, P-YC, W-JT, C-LL, KL, and H-JC analyzed data. S-AC, J-HC, C-CT, and H-KW wrote the paper. All authors contributed to the article and approved the submitted version.

## Conflict of Interest

The authors declare that the research was conducted in the absence of any commercial or financial relationships that could be construed as a potential conflict of interest.

## Abbreviations

HR: hazard ratio
CI: confidence interval
LHID2000: Longitudinal Health Insurance Database
NHIRD: National Health Insurance Research Database
ICD-9-CM: International Classification of Diseases, Ninth Revision, Clinical Modification
AD: Alzheimer's disease
VD: Vascular dementia.

## References

[1] Gorelick PB Nyenhuis D. Stroke and cognitive decline. JAMA. (2015) 314:29–30. 10.1001/jama.2015.7149 26151263

[2] Van Rooij FG Kessels RP Richard E De Leeuw FE van Dijk EJ. Cognitive impairment in transient ischemic attack patients: a systematic review. Cerebrovasc Dis. (2016) 42:1–9. 10.1159/000444282 26886189

[3] Prince M Guerchet M Prina M. The Epidemiology and Impact of Dementia: Current State and Future Trends. Geneva: World Health Organization (2015).

[4] Henon H Pasquier F Leys D. Poststroke dementia. Cerebrovasc Dis. (2006) 22:61–70. 10.1159/000092923 16645268

[5] Venkat P Chopp M Chen J. Models and mechanisms of vascular dementia. Exp Neurol. (2015) 272:97–108. 10.1016/j.expneurol.2015.05.006 25987538 PMC4631710

[6] Lu L Zhang XG Zhong LL. Acupuncture for neurogenesis in experimental ischemic stroke: a systematic review and meta-analysis. Sci Rep. (2016) 6:19521. 10.1038/srep19521 26786869 PMC4726177

[7] Seitz RJ Donnan GA. Recovery potential after acute stroke. Front Neurol. (2015) 6:238. 10.3389/fneur.2015.00238 26617568 PMC4641432

[8] Iadecola C. The pathobiology of vascular dementia. Neuron. (2013) 80:844–66. 10.1016/j.neuron.2013.10.008 24267647 PMC3842016

[9] Huang W Kutner N Bliwise DL. A systematic review of the effects of acupuncture in treating insomnia. Sleep Med Rev. (2009) 13:73–104. 10.1016/j.smrv.2008.04.002 19097814

[10] Shi GX Liu CZ Guan W Wang ZK Wang L Xiao C. Effects of acupuncture on Chinese medicine syndromes of vascular dementia. Chin J Integr Med. (2014) 20:661–6. 10.1007/s11655-013-1323-4 24155069

[11] Shi GX Li QQ Yang BF Liu Y Guan LP Wu MM. Acupuncture for vascular dementia: a pragmatic randomized clinical trial. Sci World J. (2015) 2015:161439. 10.1155/2015/161439 26495416 PMC4606072

[12] Wu P Mills E Moher D Selly D. Acupuncture in poststroke rehabilitation: a systematic review and meta-analysis of randomizedtrials. Stroke. (2010) 41:e171–9. 10.1161/STROKEAHA.109.573576 20167912

[13] Zhang JH Wang D Liu M. Overview of systematic reviews and meta-analyses of acupuncture for stroke. Neuroepidemiology. (2014) 42:50–8. 10.1159/000355435 24356063

[14] Ye Y Zhu W Wang XR Yang JW Xiao LY Liu Y. Mechanisms of acupuncture on vascular dementia-a review of animal studies. Neurochem Int. (2017) 107:204–10. 10.1016/j.neuint.2016.12.001 28034725

[15] Chon TY Lee MC. Acupuncture. Mayo Clin Proc. (2013) 88:1141–6. 10.1016/j.mayocp.2013.06.009 24079683

[16] Barnes PM Bloom B Nahin RL. Complementary and alternative medicine use among adults and children: united States, 2007. Natl Health Stat Rep. (2008) 12:1–23. 10.1037/e623942009-001 19361005

[17] Hatano S. Expperience from a multicertre stroke register: a preliminary report. Bull World Health Organ. (1976) 54:541–53. 1088404 PMC2366492

[18] Feigin VL Theadom A Barker-Collo S Starkey NJ McPherson K Kahan M. Incidence of traumatic brain injury in New Zealand: a population-based study. Lancet Neurol. (2013) 12:53–64. 10.1016/S1474-4422(12)70262-4 23177532

[19] Gardner RC Burke JF Nettiksimmons J Kaup A Barnes DE Yaffe K. Dementia risk after traumatic brain injury vs. nonbrain trauma: the role of age and severity. JAMA Neurol. (2014) 71:1490–7. 10.1001/jamaneurol.2014.2668 25347255 PMC4318558

[20] Pendlebury ST. Dementia in patients hospitalized with stroke: rates, time course, and clinico-pathologic factors. Int J Stroke. (2012) 7:570–81. 10.1111/j.1747-4949.2012.00837.x 22781124

[21] Douiri A McKevitt C Emmett ES Rudd AG Wolfe CD. Long-term effects of secondary prevention on cognitive function in stroke patients. Circulation. (2013) 128:1341–8. 10.1161/CIRCULATIONAHA.113.002236 23935013

[22] Woods B Aguirre E Spector AE Orrell M. Cognitive stimulation to improve cognitive functioning in people with dementia. Cochrane Database Syst Rev. (2012) 2:CD005562. 10.1002/14651858.CD005562.pub2 22336813

[23] Zhao ZQ. Neural mechanism underlying acupuncture analgesia. Prog Neurobiol. (2008) 85:355–75. 10.1016/j.pneurobio.2008.05.004 18582529

[24] Ye Y Li H Yang JW Wang XR Shi GX Yan CQ. Acupuncture attenuated vascular dementia-induced hippocampal long-term potentiation impairments *via* activation of D1/D5 receptors. Stroke. (2017) 48:1044–51. 10.1161/STROKEAHA.116.014696 28289242

[25] Li CH Chang YH Chou MC Chen CH Ho BL Hsieh SW. Factors of post-stroke dementia: a nationwide cohort study in Taiwan. Geriatr Gerontol Int. (2019) 19:815–22. 10.1111/ggi.13725 31267646

[26] Pasquier F Hénon H Leys D. Risk factors and mechanisms of post-stroke dementia. Rev Neurol. (1999) 155:749–53. 10528361

[27] Pendlebury ST Rothwell PM. Prevalence, incidence, and factors associated with pre-stroke and post-stroke dementia: a systematic review and meta-analysis. Lancet Neurol. (2009) 8:1006–18. 10.1016/S1474-4422(09)70236-4 19782001

[28] Mijajlović MD Pavlović A Brainin M Heiss WD Quinn TJ Ihle-Hansen HB. Post-stroke dementia—a comprehensive review. BMC Med. (2017) 15:11. 10.1186/s12916-017-0779-7 28095900 PMC5241961

[29] Seshadri S Wolf PA. Lifetime risk of stroke and dementia: current concepts, and estimates from the Framingham study. Lancet. (2007) 3:1106–14. 10.1016/S1474-4422(07)70291-0 18031707

[30] Yang Z Edwards D Burgess S Brayne C Mant J. Association of prior atherosclerotic cardiovascular disease with dementia after stroke: a retrospective cohort study. J Alzheimers Dis. (2020) 77:1157–67. 10.3233/JAD-200536 32925051 PMC7683071

[31] Pendlebury ST Rothwell PM Oxford Vascular Study. Incidence and prevalence of dementia associated with transient ischaemic attack and stroke: analysis of the population-based Oxford Vascular Study. Lancet Neurol. (2019) 18:248–58. 10.1016/S1474-4422(18)30442-3 30784556 PMC6390174

[32] Cai W Zhang K Li P Zhu L Xu J Yang B. Dysfunction of the neurovascular unit in ischemic stroke and neurodegenerative diseases: an aging effect. Ageing Res Rev. (2017) 34:77–87. 10.1016/j.arr.2016.09.006 27697546 PMC5384332

[33] Ballard C Gauthier S Corbett A Brayne C Aarsland D Jones E. Alzheimer's disease. Lancet. (2011) 377:1019–31. 10.1016/S0140-6736(10)61349-9 21371747

[34] Shih CC Hsu YT Wang HH Chen TL Tsai CC Lane HL. Decreased risk of stroke in patients with traumatic brain injury receiving acupuncture treatment: a population-based retrospective cohort study. PLoS ONE. (2014) 9:e89208. 10.1371/journal.pone.0089208 24586597 PMC3929662

[35] Wang HK Huang CY Sun YT Li JY Chen CH Sun Y. Smoking paradox in stroke survivors? Uncovering the truth by interpreting 2 sets of data. Stroke. (2020) 51:1248–56. 10.1161/STROKEAHA.119.027012 32151234

[36] Lin CK Chen PY Wu YY Wu CC Chen HJ Liang CL. Adjunctive statin therapy reduces mortality after acute hemorrhagic stroke. Risk Manag Healthc Policy. (2021) 14:177–83. 10.2147/RMHP.S290964 33488130 PMC7814233

